# Supplementary material for: Exploring microbial diversity and biosynthetic potential in zoo and wildlife animal microbiomes
Source: Nat Commun. 2024 Sep 26;15:8263. doi: 10.1038/s41467-024-52669-9 (PMC11427580; doi:10.1038/s41467-024-52669-9)
Supplement: Supplementary file 1 — Supplementary Information [file 41467_2024_52669_MOESM1_ESM.pdf]

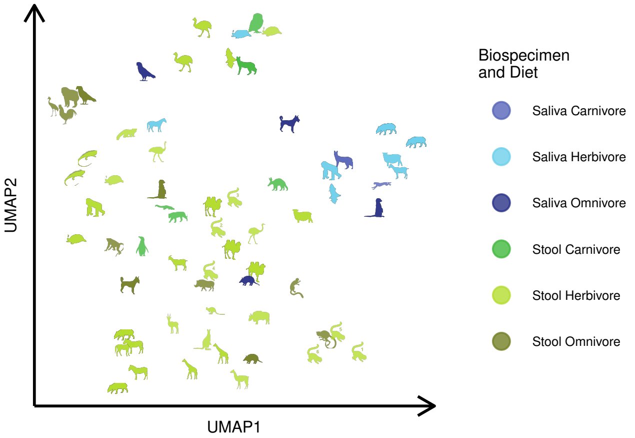

Supplementary Figure 1, Ordination analysis: Two-dimensional embedding of the dataset generated with UMAP computed on FracMinHash dissimilarities. Silhouettes represent the different animals as depicted in Figure 1c. Colors represent the different specimen and diet combinations.

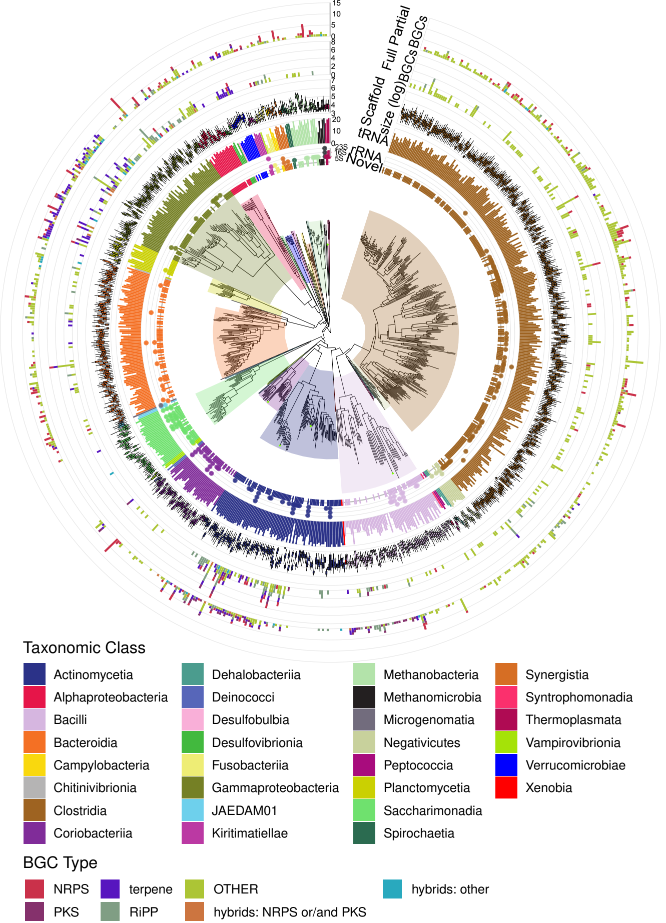

Supplementary Figure 2, SGBs: Complete version of Figure 2a. Visualization of dereplicated SGBs with color-encoded BGC and class information.

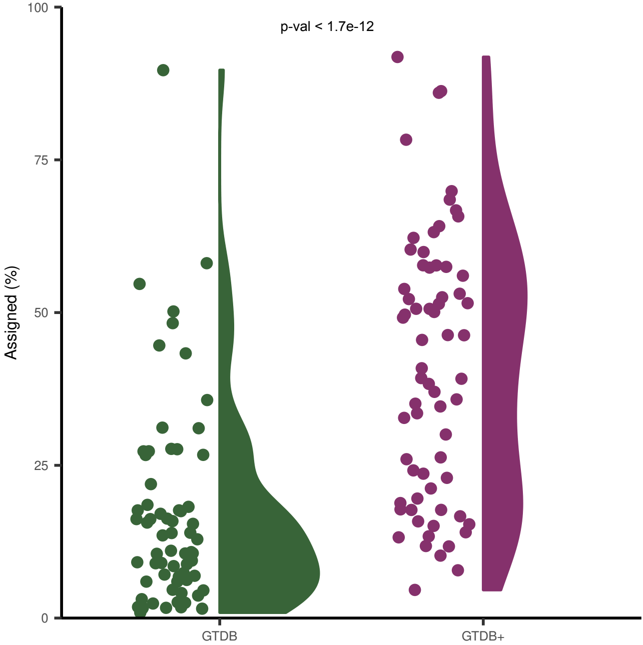

Supplementary Figure 3, Assignment rate: Relative amount of reads after quality control assigned during taxonomic profiling using GTDB and GTDB extended by our SGBs respectively. The indicated p-value is the statistical significance of a paired two-sided Wilcoxon rank sum test (n = 66).

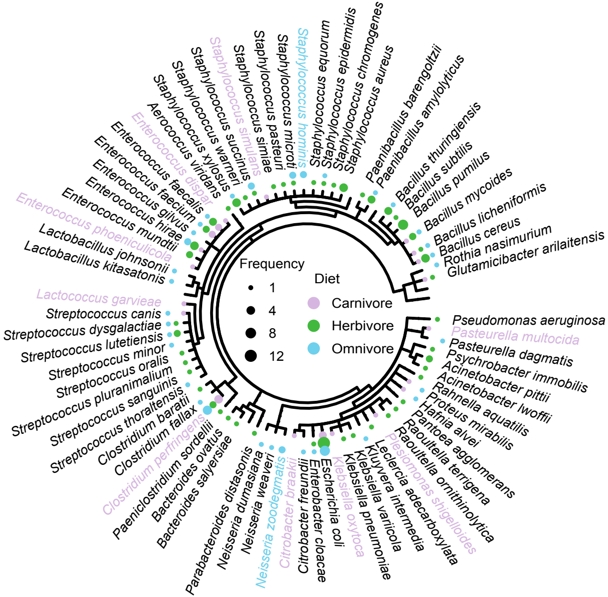

Supplementary Figure 4, Mass spectrometry: Bacterial species identified by performing mass spectrometry after culturing in different mediums. The bacterial taxonomic classes are provided as a tree structure. Counts indicate in how many different animals the associated bacterial species was detected.

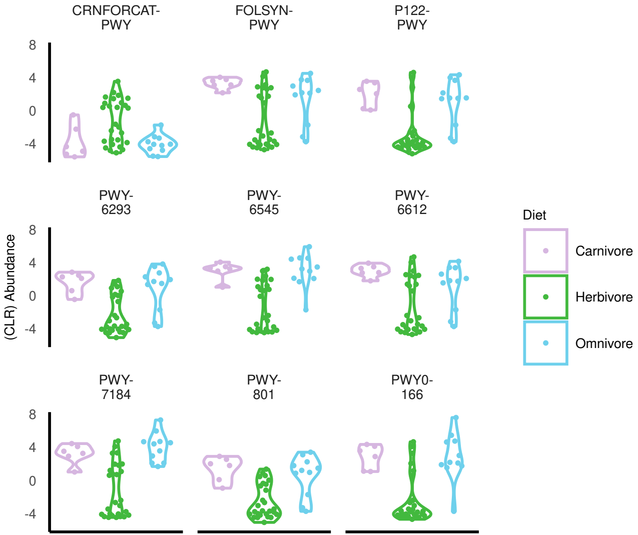

Supplementary Figure 5, Differential pathway abundance: Statistically significant results of the differential pathway abundance analysis with ANCOMBC [82] after the Benjamini-Hochberg p-value adjustment. During analysis omnivores and carnivores were agglomerated and compared against herbivores (n = 44).

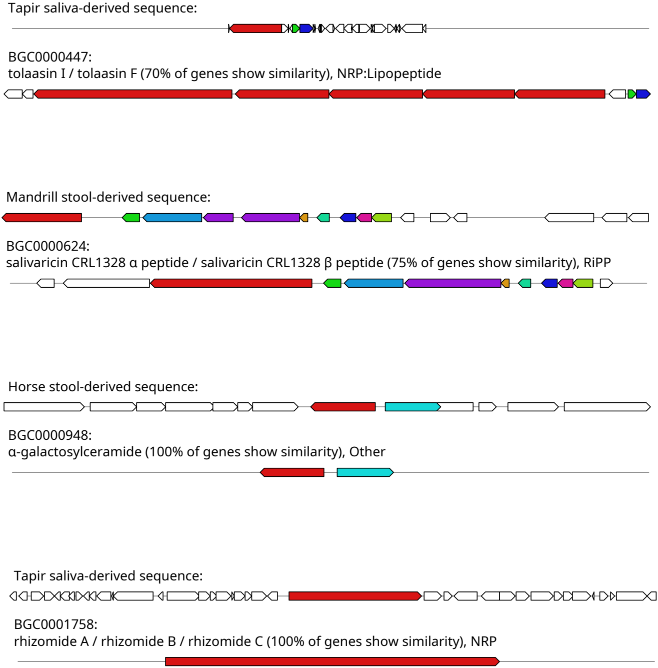

Supplementary Figure 6, Known BGCs: Selection of detected BGCs with a >75% similarity to MIBiG annotated clusters. Comparisons are minor adaptations of the figures directly reported by antiSMASH.

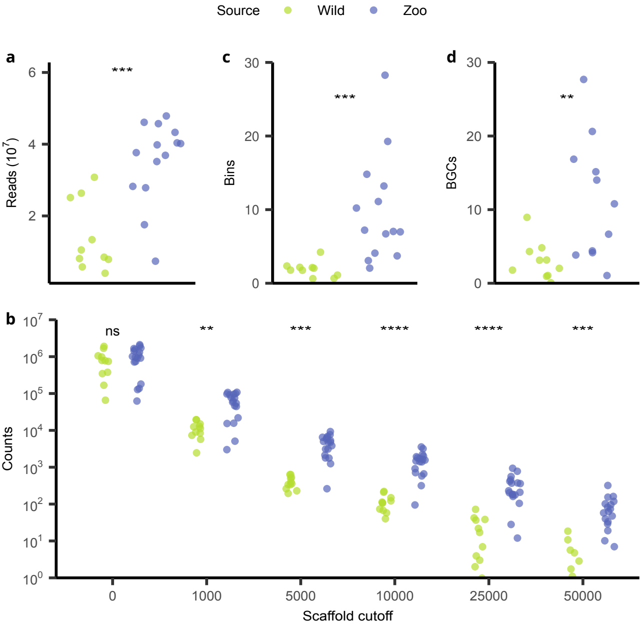

Supplementary Figure 7, Study QC Comparison: Detailed comparison of the matched samples in our dataset and the dataset of Youngblut et al. (35). Two-sided Wilcoxon rank sum tests were performed to estimate statistical significance. **a)** Number of reads after host DNA removal and quality control. **b)** Contig length distribution after metagenomic assembly. **c)** Number of SGBs generated with the two dataset subsets. **d)** Number of BGCs predicted from each initial input sample (n = 26).
