# Supplementary material for: Exploring microbial diversity and biosynthetic potential in zoo and wildlife animal microbiomes
Source: Nat Commun. 2024 Sep 26;15:8263. doi: 10.1038/s41467-024-52669-9 (PMC11427580; doi:10.1038/s41467-024-52669-9)
Supplement: Supplementary file 3 — Description of Additional Supplementary Files [file 41467_2024_52669_MOESM3_ESM.pdf]

Description of Additional Supplementary Files

File Name: Supplementary Data 1
Description: Metadata: Table aggregating metadata of the different samples including animal species, reference genome used for decontamination, suitability of the reference, specimen, relative loss during host decontamination, and the assigned diet label for data analysis.

File Name: Supplementary Data 2
Description: SGB data: Information on each dereplicated SGB that matched at least medium MIMAG quality. Displayed information includes rRNA, tRNA, and scaffold counts as well as completeness, contamination, and overall genome size for quality information. Further two classification schemes are provided. First, GTDB lineage as provided by GTDB-Tk is given. Second, a best-matching NCBI taxonomy classification is provided.

File Name: Supplementary Data 3
Description: Mass spectrometry results: Aggregated results of the mass spectrometry data. Providing an overview of presence by diet and specimen type for each bacterial species on the first sheet. The second sheet lists each unique bacterial species – zoo sample combination that was detected.

File Name: Supplementary Data 4
Description: BGC summary: Overview data of the observed partial and full BGCs including positional, type, and location SGB information.

File Name: Supplementary Data 5
Description: AMR genes overview: The first sheet lists all predicted AMR genes of three different tools. The second sheet indicates our manually added Ambler classification information for the ResFinder and AMRFinderPlus results.
